# Horizontal transfer between loose compartments stabilizes replication of fragmented ribozymes

**DOI:** 10.1371/journal.pcbi.1007094

**Published:** 2019-06-06

**Authors:** Atsushi Kamimura, Yoshiya J. Matsubara, Kunihiko Kaneko, Nobuto Takeuchi

**Affiliations:** 1 Department of Basic Science, Graduate School of Arts and Sciences, The University of Tokyo, Komaba, Meguro-ku, Tokyo, Japan; 2 Research Center for Complex Systems Biology, Universal Biology Institute, The University of Tokyo, Komaba, Meguro-ku, Tokyo, Japan; 3 School of Biological Sciences, The University of Auckland, Private Bag, Auckland, New Zealand; University of California Irvine, UNITED STATES

## Abstract

The emergence of replicases that can replicate themselves is a central issue in the origin of life. Recent experiments suggest that such replicases can be realized if an RNA polymerase ribozyme is divided into fragments short enough to be replicable by the ribozyme and if these fragments self-assemble into a functional ribozyme. However, the continued self-replication of such replicases requires that the production of every essential fragment be balanced and sustained. Here, we use mathematical modeling to investigate whether and under what conditions fragmented replicases achieve continued self-replication. We first show that under a simple batch condition, the replicases fail to display continued self-replication owing to positive feedback inherent in these replicases. This positive feedback inevitably biases replication toward a subset of fragments, so that the replicases eventually fail to sustain the production of all essential fragments. We then show that this inherent instability can be resolved by small rates of random content exchange between loose compartments (i.e., horizontal transfer). In this case, the balanced production of all fragments is achieved through negative frequency-dependent selection operating in the population dynamics of compartments. The horizontal transfer also ensures the presence of all essential fragments in each compartment, sustaining self-replication. Taken together, our results underline compartmentalization and horizontal transfer in the origin of the first self-replicating replicases.

## Author summary

How evolution got started is a crucial question in the origin of life. One possibility is that RNA molecules gained the ability to catalyze self-replication. Researchers recently proposed how this possibility might have been realized: a long RNA catalyst was divided into short replicable fragments, and these fragments self-assembled into the original long catalyst. Ingenious though it is, we expose a hidden flaw in this proposal. An auto-catalytic system based on fragmented catalysts involves positive feedback, which necessarily biases replication toward specific fragments and eventually halts the replication of the whole system. However, we also propose an effective remedy to this flaw: compartmentalization and content exchange among compartments generate negative feedback, which tightly coordinates the replication of different fragments.

## Introduction

One of the crucial questions in the origin of life is how molecules acquired the capability of undergoing open-ended Darwinian evolution [1, 2]. A potential answer is offered by the template-directed self-replication of a replicase, a replicase that can replicate itself. To determine whether such self-replication is demonstrable in RNA, considerable effort has been devoted to the artificial evolution of RNA polymerase ribozymes [3–10]. A recent milestone in this effort is the demonstration of ‘riboPCR,’ the exponential amplification of RNA through a PCR-like mechanism catalyzed entirely by RNA [8]. The glaring issue, however, has been that the replicases synthesized so far have limitations in processivity and fidelity, so that they can replicate only oligomers much shorter than themselves (or long unstructured cytidine-rich polymers, which exclude the ribozymes themselves).

As a potential solution to this problem, Mutschler et al. and Horning et al. have recently proposed the fragmentation and self-assembly of a replicase. According to their proposals, a replicase is fragmented into multiple sequences that are short enough to be replicable by the replicase and, moreover, capable of self-assembling into a functional replicase [7, 9]. The possibility of reconstituting a functional ribozyme from its fragments through self-assembly has been experimentally demonstrated [7, 9, 10], attesting the chemical plausibility of the proposals.

However, the exponential amplification of multiple distinct fragments raises a question about the dynamical stability of the proposed autocatalytic system. The continued replication of fragmented replicases requires the sustained production of all its essential fragments in yields proportional to the stoichiometric ratio of the fragments in a replicase [11–13]. However, each fragment is replicated by the replicase and thus grows exponentially. If some fragments was replicated persistently faster than the others, the former would out-compete the latter, causing a loss of some essential fragments and hence the cessation of self-replication.

The above consideration led us to examine whether and under what conditions fragmented replicases achieve continued self-replication. Using mathematical modeling, we discovered that the fragmented replicases fail to display continued self-replication under a simple batch condition. Replication is inevitably biased toward a subset of the fragments owing to positive feedback inherent in the replication cycle of the fragmented replicases, and the loss of fragment diversity eventually halts self-replication.

To find a way to resolve the above instability, we next examined the role of compartmentalization. Our model assumes a population of protocells (primitive cells; hereinafter referred to as “cells”), each encapsulating a finite number of fragments and replicases. We found that compartmentalization, in principle, allows the continued self-replication of the replicases by the stochastic correction mechanism [14, 15]. This mechanism selects fast-growing cells with a better combination of the fragments by removing cells that cannot grow or grow only slowly. We found, however, that there is severe restriction to the number of molecules in a cell and to the number of cells, for this mechanism to work. Indeed the stochastic correction mechanism works only if the number of fragments per cell is not large and if a sufficient number of cells exists. Furthermore, with this selection, a large number of cells are discarded, and a large number of fragments is thrown out without producing functional replicases. Hence, we need some other factors beyond the stochastic correction mechanism in order to realize a robust and effective replication system.

Finally, we show that horizontal transfer between cells provides an effective mechanism for the continued replication of the fragmented ribozymes. One may naively expect that such horizontal transfer impedes the stochastic correction mechanism because by molecule exchange between cells each compartment is not perfectly separated (however, see [16] for a contrasting expectation). Therefore, horizontal transfer might be expected to be detrimental to the continued self-replication of the fragmented replicases. On the contrary, we found that the horizontal transfer of intermediate frequencies substantially stabilizes the system to such an extent that the parameter constraints imposed by the stochastic correction mechanism are almost completely removed.

## Model

We consider the simplest model of fragmented replicases, in which a catalyst consists of two fragments. The fragments (denoted by *X* and *Y*) self-assemble into the catalyst (denoted by *C*), and the catalyst disassembles into the fragments as follows:

$$\begin{array}{c} X+Y\overset{k^{f}}{\to }C,\;C\overset{k^{b}}{\to }X+Y. \end{array}$$ (1)

We assume that the catalytst cannot replicate its own copies, but can replicate its fragments because shorter templates are more amenable to ribozyme-catalyzed replication as mentioned above. Therefore,

$$\begin{array}{c} X+C\overset{k_{x}}{\to }2X+C, \end{array}$$ (2)

$$\begin{array}{c} \;Y+C\overset{k_{y}}{\to }2Y+C, \end{array}$$ (3)

where the monomers are ignored under the assumption that their concentrations are buffered at constant levels, and complementary replication is ignored for simplicity. In the presence of the catalyst, each fragment replicates at a rate proportional to its copy number. Hence, the fragments undergo exponential amplification.

## Results

### Failure of balanced replication of fragments under a batch condition

First, we show that the replication of the fragments *X* and *Y* are unstable in a batch condition: replication is biased toward either of the fragments even if the rate constants for *X* and *Y* are identical, and the minor fragment is gradually diluted out from the system, so that the replication of the catalysts eventually stops. In this paper, we mainly focus on the situation where the rate constants are equal (*k*_*x*_ = *k*_*y*_ = *k*) because our results remain qualitatively the same as long as the difference between *k*_*x*_ and *k*_*y*_ is sufficiently small.

We assume that the reactions undergo in a well-mixed batch condition so that the dynamics of the concentrations of *X*, *Y*, and *C* (denoted by *x*, *y*, and *c*, respectively) are written as follows:

$$\frac{dx}{dt}=(-k^{f}xy+k^{b}c+kxc)-x\mu ,$$ (4)

$$\frac{dy}{dt}=(-k^{f}yx+k^{b}c+kyc)-y\mu ,$$ (5)

$$\frac{dc}{dt}=(k^{f}xy-k^{b}c)-c\mu ,$$ (6)

where *μ* = *k*(*x* + *y*)*c*. In the right-hand side of these equations, the first terms in the brackets represent chemical reactions, and the second terms multiplied by *μ* represent dilution. The dilution terms are defined so as to keep the total molecular mass *x* + *y* + 2*c* constant (the dilution terms could alternatively be defined as *μ* = −*k*^*f*^*xy* + *k*^*b*^*c* + *k*(*x* + *y*)*c*, in which case the total concentration *x* + *y* + *c*, rather than the total molecular mass *x* + *y* + 2*c*, would be kept constant; however, results would not essentially change). Within the brackets enclosing the reaction terms, the first and second terms represent forward and backward reactions of (1), respectively. The third terms, which are present only in Eqs (4) and (5), denote the replication of *X* and *Y* through reactions (2) and (3), respectively.

By introducing variables *x*_*tot*_ = *x* + *c* and *y*_*tot*_ = *y* + *c*, one can write

$$\begin{array}{c} \frac{d}{dt}(\frac{x_{tot}}{y_{tot}})=\frac{kc^{2}}{y_{tot}^{2}}(x_{tot}-y_{tot}). \end{array}$$ (7)

This equation indicates that a steady-state solution satisfies *x*_*tot*_ = *y*_*tot*_. This solution is, however, unstable: a small increase in, say, *x*_*tot*_ over *y*_*tot*_ gets amplified because $kc^{2}/y_{tot}^{2}$ is always positive, and, as a consequence, replication is biased to *X*. Intuitively, when *x*_*tot*_ is slightly greater than *y*_*tot*_, the amount of free fragments *x* must also be greater than *y* because the same amount of *X* and *Y* are incorporated into catalysts. Therefore, the replication of *X* occurs more frequently than that of *Y* because more templates of *X* are available. As a result, the increase of *x*_*tot*_ is greater than that of *y*_*tot*_. Because of this positive feedback, the concentration of the minor fragment *Y* gradually decreases as it is diluted out from the system, and, as a consequence, that of the catalysts *C* also decreases. Finally, the replication reaction stops once the catalysts are lost from the system.

The instability of replication under a batch condition can be generally demonstrated for catalysts composed of an arbitrary number of fragments by straightforwardly extending the above model (S1 Text).

### Compartmentalization can overcome the unstable replication by selecting out non-growing cells but only under strong constraints on the sizes of cell volume and population

**Fig 1:**
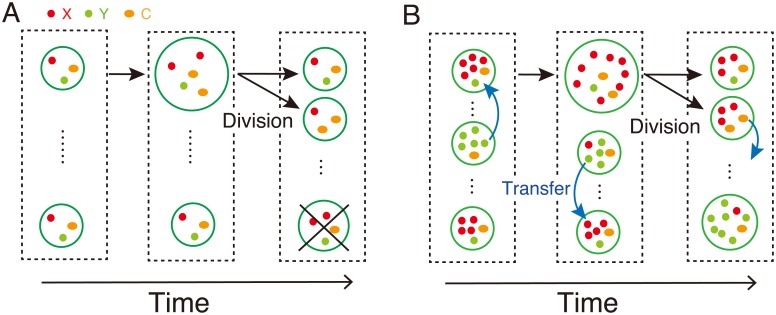
Schematic of the model. (A) The fragments *X*, *Y*, and their catalysts *C* are encapsulated into *N*_*cell*_ cells, and they undergo the reactions (1) to (3) to increase their volume. When the volume of a cell exceeds a threshold *V*_*Div*_, the cell divides and the components inside are randomly partitioned into two daughter cells. At the same time, one randomly-chosen cell is removed to fix the total number of cells. (B) With the transfer, the components are transferred among the cells [denoted by blue arrows] with a transfer constant *D* through the time-course of the model shown in (A).

The introduction of compartments and their competitions can overcome the unstable replication. When the system is compartmentalized into a number of cells, stochasticity in cell compositions, competition for growth and division of cells provide a possible solution to avoid the loss of fragments: As the cells grow and eventually split into two with fragments distributed randomly between the daughter cells, cells with both *X* and *Y* fragments continue growth, while cells without either of them cannot grow. By introducing such a stochastic correction mechanism at the cell level [14], one expects that the instability by the positive feedback at the molecule level can be resolved. To investigate this, we assume that the fragments and their assembly to function as a catalyst are partitioned into *N*_*cell*_ cells: the reactions occur in each cell (Fig 1A). We adopted stochastic simulation using Gillespie algorithm [17] for reactions (1)–(3). We assume that the volume of each cell is proportional to the number of fragments inside, and as the number of fragments increases in a cell, the cell grows. When the total number of fragments reaches a threshold *V*_*Div*_, the cell divides with the components randomly partitioned into two daughter cells. Here, at the division event, one randomly-chosen cell is removed to fix the total number of cells *N*_*cell*_. By this cell-cell competition, cells with biased composition of *X* and *Y* are selected out because their growth is slow.

**Fig 2:**
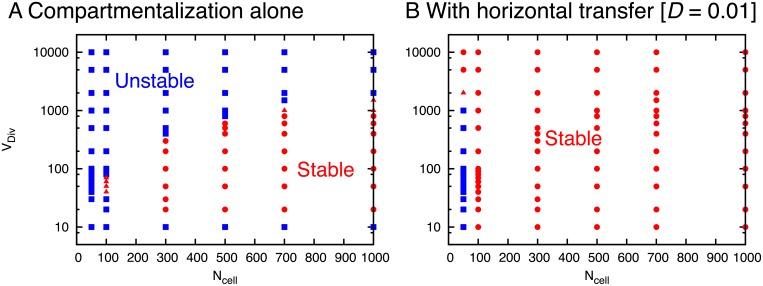
Sets of division threshold *V*_*Div*_ and the number of cells *N*_*cell*_ with which the unstable replication of reactions (1)–(3) is avoided by (A) compartmentalization alone and (B) that with horizontal transfer of the transfer constant *D* = 0.01. For the sets shown as stable [red circles], the system can continuously have cells with both fragments in the simulations up to 4 × 10^5^ division events from an initial condition where *V*_*Div*_/4 copies of each *X* and *Y* are in each cell. For the sets shown as unstable [blue squares], all cells with both fragments are lost from the system and it cannot continue growth. For the sets located at the boundary of stable and unstable area [shown in red triangles], the outcome depends on simulation runs.

The relevant parameters for controlling the effect of compartmentalization are the division threshold *V*_*Div*_ and the number of cells *N*_*cell*_. Fig 2A shows sets of the parameters with which the stochastic correction mechanism can avoid the unstable replication, by suppressing the positive feedback and selecting cells keeping both fragments. If *V*_*Div*_ is very small (of the order of 10), the stochasticity of cell components is too strong to maintain both fragments continuously and either of them is lost for all cells. Hence, the system cannot continue growth. On the other hand, if *V*_*Div*_ is too large, stochasticity in components decreases. In each cell, the balance of fragments is broken, and the replication is biased to either of *X* or *Y*. Then, components of each cell are dominated by either of free *X* or *Y*, and the number of catalysts in dividing cells gradually decreases to one because at least one catalyst is necessary to replicate fragments (Note that the cells with a single catalyst can divide but cannot grow because the cells cannot make two of itself). Even when the *N*_*cell*_ cells are separated into the equal number of *X*-dominant and *Y*-dominant cells, the stochastic correction mechanism does not work because *V*_*Div*_ is too large. Thus, the random drift will finally result in bias to either of *X*-dominant or *Y*-dominant cells. By division events, daughter cells without catalysts randomly replace remaining cells, therefore, the cells with catalysts are finally removed from the system.

For values of *V*_*Div*_ in-between, some of *N*_*cell*_ cells keep both *X* and *Y*, and can continue the replication. Besides *V*_*Div*_, the number of cells *N*_*cell*_ is also restricted, to maintain such cells keeping both fragments *X* and *Y*. At division events, dividing cells without both fragments randomly replace remaining cells. Hence, when the number of cells *N*_*cell*_ is small, all the cells with both fragments will be finally removed. As the number of cells *N*_*cell*_ increases, the probability that all the cells with both fragments are removed decreases. As a result, the range of *V*_*Div*_ with stable replication increases. Note that the above mechanism is based on the selection of fast-growing cells, and a number of fragments are thrown out with the removal of cells, although they are still functional if combined across the cells.

### Horizontal transfer of fragments with small rates removes the constraints of compartments for stable replication

Without the selection in cell population nor the restriction to *V*_*Div*_, horizontal transfer of fragments between cells rescues the loss of fragments and enables continuous replication by maintaining the balance between *X* and *Y*. If the *X*-dominant and *Y*-dominant cells coexist in the cell population, the transfer between cells avoids loss of fragments for both cells by supplying fragments to each other because each fragment is in excess for cells on one side but lacking for cells on the other side.

For the purpose, we consider random mutual transfers of molecules among the *N*_*cell*_ cells (Fig 1B). To implement the transfer, we consider reactions, $X\overset{D}{\to }0$, $Y\overset{D}{\to }0$, $C\overset{D}{\to }0$ so that the *X*, *Y* and *C* are removed from a cell, respectively, with rate in proportional to each concentration, i.e., *Dx*, *Dy*, and *Dc*. This gives diffusion out of the cell. At the same time, the component is added to another randomly-chosen cell. The results are not sensitive even if the fragments are partially lost in this process (S2 Text).

**Fig 3:**
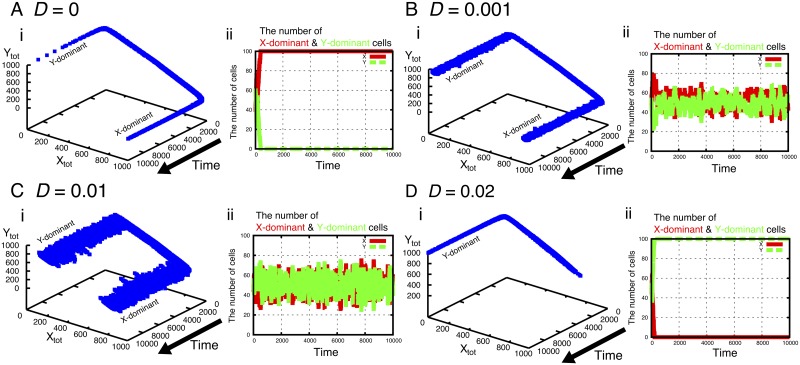
(i) The number of fragments *X*_*tot*_ and *Y*_*tot*_ of dividing cells and (ii) the number of *X*-dominant and *Y*-dominant cells at corresponding time for the transfer constants (A) *D* = 0 (B) *D* = 0.001 (C) *D* = 0.01 and (D) *D* = 0.02. Initially, the numbers of *X*_*tot*_ and *Y*_*tot*_ are approximately equal and, as time goes on, cells are differentiated into either of *X*-dominant or *Y*-dominant compositions. For *D* = 0 (A), the system is unstable: only *X*-dominant cells (for this run) dominate (ii) and finally, cells cannot continue growth. For *D* = 0.001 (B) and 0.01 (C), the system is stable; *X* and *Y* fragments coexist in each cell with unequal population (i). Here, the asymmetry between the major and minor fragments gets smaller as *D* increases. In addition, the two types of cells, *X*-dominant and *Y*-dominant cells coexist with the equal population (ii). As *D* increases further [*D* = 0.02 (D)], the system gets unstable and only either of *X* or *Y* remains (ii). The parameters are *V*_*Div*_ = 1000, *N*_*cell*_ = 100, *k*^*f*^ = *k*^*b*^ = 1, and *k*_*x*_ = *k*_*y*_ = 1.

With the transfer among cells, the replication of the fragments is stabilized when the transfer constant *D* is small. In fact, the constraints of *V*_*Div*_ and *N*_*cell*_ are drastically eliminated (Fig 2B). As long as the parameters are not extremely small, the stable replication continues. For small positive values of *D* (Fig 3B and 3C), the cell keeps on growing with the coexistence of *X* and *Y* molecules in each cell, even for large *V*_*Div*_ where only *X*-dominant or *Y*-dominant cells remain for *D* = 0 (Fig 3A). Here, the asymmetry between the fractions of the major and minor fragments gets smaller as *D* increases. In addition, two types of cells, *X*-dominant and *Y*-dominant cells coexist roughly with equal population (Fig 3B(ii) and 3C(ii)). As *D* is increased further (Fig 3D), the system gets unstable and only either of *X* or *Y* remains. This is natural, because for a large *D* limit, the system is well mixed, and the system is reduced back to the case without compartmentalization. The above result remains valid even if *k*_*x*_ ≠ *k*_*y*_. In this case, the number ratio between X-dominant cells and Y-dominant cells changes to compensate for the difference between *k*_*x*_ and *k*_*y*_ (S3 Text).

### Bifurcation explains the stable replication with small rates of horizontal transfer in two subsystems as an approximation of cell population

To answer why the small rates of transfer stabilizes the system, we approximate the dynamics of the population of cells by considering the dynamics of two subsystems between which the fragments are transferred. We assume an equal population of *X*-dominant and *Y*-dominant cells as two subsystems of an equal volume, denoted as subsystem 1 and 2, respectively. We write the total concentration of *X* (the total of free *X*s and *C*s) of each subsystem as $x_{tot}^{1}$ and $x_{tot}^{2}$, and the total of *Y* as $y_{tot}^{1}$ and $y_{tot}^{2}$. Likewise, the concentration of free *X*, free *Y*, and *C* are denoted as *x*^*i*^, *y*^*i*^, and *c*^*i*^, respectively.

In each of the subsystem *i* (*i* = 1, 2), the dynamics of the reactions is written as Eqs (4) to (6). Then, the time-derivative of the variable $x_{tot}^{i}=x^{i}+c^{i}$ is obtained by adding both sides of Eqs (4) and (6) as,

$$\begin{array}{cc} \frac{dx_{tot}^{i}}{dt}=\frac{d(x^{i}+c^{i})}{dt} & =kx^{i}c^{i}-\left(x^{i}+c^{i}\right)\mu_{i},\\ & =k\left(x_{tot}^{i}-c^{i}\right)c^{i}-x_{tot}^{i}\mu_{i}, \end{array}$$ (8)

where we assume *k*_*x*_ = *k*_*y*_ = *k*. Likewise, the time-derivative of $y_{tot}^{i}$ is obtained by adding both sides of Eqs (5) and (6) as

$$\begin{array}{cc} \frac{dy_{tot}^{i}}{dt}=\frac{d(y^{i}+c^{i})}{dt} & =ky^{i}c^{i}-\left(y^{i}+c^{i}\right)\mu_{i},\\ & =k\left(y_{tot}^{i}-c^{i}\right)c^{i}-y_{tot}^{i}\mu_{i}. \end{array}$$ (9)

The dilution rate *μ*^*i*^ is defined as

$$\begin{array}{c} \mu^{i}=k\left(x^{i}+y^{i}\right)c^{i}=kx^{i}c^{i}+ky^{i}c^{i}=k\left(x_{tot}^{i}-c^{i}\right)c^{i}+k\left(y_{tot}^{i}-c^{i}\right)c^{i}=k\left(1-2c^{i}\right)c^{i}, \end{array}$$

so that the total concentration $x_{tot}^{i}+y_{tot}^{i}$ is kept at one. The above definition of *μ*_*i*_ is obtained by setting the sum of the right-hand-sides of Eqs (8) and (9) to zero and $x_{tot}^{i}+y_{tot}^{i}$ to one. This dilution corresponds to the decrease of concentrations due to the volume growth of a subsystem. In this section, we assume that the volumes of the two subsystems are kept identical to each other, ignoring the dynamics of the volumes (in the next section, we relax this assumption and investigate a mechanism that maintains the balance of the volumes).

In addition to the reactions, the components are transfered between the subsystems. Thus, the changes of $x_{tot}^{i}$ are written as

$${\dot{x}}_{tot}^{1}=\frac{dx_{tot}^{1}}{dt}=F^{1}-\frac{D}{2}x_{tot}^{1}+\frac{D}{2}x_{tot}^{2},$$ (10)

$${\dot{x}}_{tot}^{2}=\frac{dx_{tot}^{2}}{dt}=F^{2}-\frac{D}{2}x_{tot}^{2}+\frac{D}{2}x_{tot}^{1}.$$ (11)

where $F^{i}=k\left(x_{tot}^{i}-c^{i}\right)c^{i}-x_{tot}^{i}\mu_{i}$ denote the right-hand-side of Eq (8). The second and third terms in Eqs (10) and (11) denote average out- and in-flow of the components *X* by the transfer, respectively. These average flows are estimated as follows: the amount of the fragment *X* diffusing out from the subsystem 1 is $Dx_{tot}^{1}$, but half of them is returned to the subsystem itself because, in our simulation, the population of cells is divided into *X*-dominant and *Y*-dominant cells with the equal population of *N*_*cell*_/2 and each fragment diffusing out from a cell is randomly re-distributed into one of the cells, i.e., half of the fragments are distributed into *X*-dominant cells. Thus, the effective amount of fragments transferred from subsystem 1 to subsystem 2 is $Dx_{tot}^{1}/2$. In the same manner, the effective amount of the fragment *X* for subsystem 1 transferred from subsystem 2 is $Dx_{tot}^{2}/2$. The diffusion terms for *Y* are obtained in the same manner.

The fixed-point solutions of Eqs (10) and (11) are analytically obtained for *k*^*f*^ = *k*^*b*^ by the following approximations. We first assume that the dynamics of reaction (1) is much faster than those of reactions (2) and (3), and transfers. Under this assumption, the condition *k*^*f*^*x*^*i*^*y*^*i*^ = *k*^*b*^*c*^*i*^ holds. For *k*^*f*^ = *k*^*b*^, the condition is re-written in terms of $x_{tot}^{i}$ and $y_{tot}^{i}$ as $\left(x_{tot}^{i}-c^{i}\right)\left(y_{tot}^{i}-c^{i}\right)=c^{i}$. Thus, the concentration of *c*^*i*^ is obtained as $c^{i}=1-\sqrt{1-x_{tot}^{i}y_{tot}^{i}}$. Moreover, because $x_{tot}^{i}$ and $y_{tot}^{i}$ are highly asymmetric, i.e., $x_{tot}^{i}y_{tot}^{i}⪡1$, as predicted by Eq (7) and confirmed in Fig 3B and 3C, we approximate *c*^*i*^ as follows: $c^{i}=1-\sqrt{1-x_{tot}^{i}y_{tot}^{i}}\approx x_{tot}^{i}y_{tot}^{i}/2$. By substituting the expression for *c*^*i*^ into Eqs (10) and (11), and solving them in a steady-state condition, $dx_{tot}^{i}/dt=0$, the stable fixed point is obtained as

$$\begin{array}{c} x_{tot}^{1}=\frac{1}{2}(1+\sqrt{1-4\sqrt{2D/k}}), \end{array}$$ (12)

and

$$\begin{array}{c} x_{tot}^{2}=\frac{1}{2}(1-\sqrt{1-4\sqrt{2D/k}}). \end{array}$$ (13)

**Fig 4:**
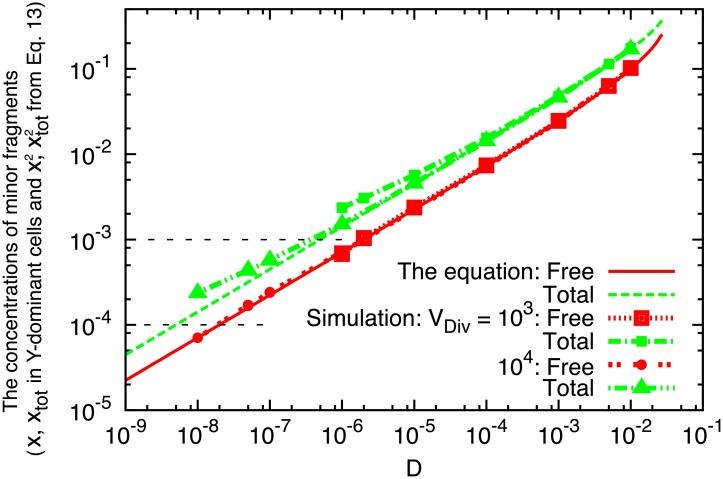
The concentrations of minor fragments *x* and *x*_*tot*_ at division events for *Y*-dominant cells as a function of *D*. Free [red] and Total [green] indicate *x* and *x*_*tot*_, respectively. For the free fragments [*x*], the results of simulations [red curves with points for *V*_*Div*_ = 10^3^ and 10^4^] agree well with the solution $x^{2}=x_{tot}^{2}-c^{2}$ from Eq (13) [red curve]. For the total fragments [*x*_*tot*_], the simulations [green curves with points for *V*_*Div*_ = 10^3^ and 10^4^] agree with the solution $x_{tot}^{2}$ of Eq (13) [green curve] for larger *D*, but shift to larger values for smaller *D*. This is because cells must possess at least one catalyst to divide, therefore, the total fragments including *c* shift to larger values as it approaches the minimum requirement. Note that $x_{tot}^{2}<1/2$, and the concentration of major fragments is obtained by $x_{tot}^{1}=1-x_{tot}^{2}$. For reference, the values of *x*_*tot*_ = 1/*V*_*Div*_ at which the number of *c* is equal to one for *V*_*Div*_ = 10^3^ and 10^4^ are shown by horizontal dotted lines, respectively. The other parameters are *N*_*cell*_ = 100, *k*^*f*^ = *k*^*b*^ = 1, and *k*_*x*_ = *k*_*y*_ = *k* = 1.

Here, we assume the dominant fragment of subsystem 1 is *X* and that of subsystem 2 is *Y*: $x_{tot}^{1}>1/2$ and $x_{tot}^{2}<1/2$. Further, only the ratio of the transfer constant *D* to the replication rate *k* matters so that we assume *k* = 1 without loss of generality. Eq (13) is compared with the results of stochastic simulations in Fig 4. For large transfer constants *D*, Eq (13) agrees well with the simulations, attesting the validity of the approximations involved in Eq (13). For small transfer constants, however, Eq (13) underestimates the total concentration of the minor fragment $x_{tot}^{2}$ in the simulations. This underestimation is due to the fact that in the simulations, cells must possess at least one catalyst molecule to divide; that is, $x_{tot}^{2}$ cannot decrease below 1/*V*_*Div*_. The presence of such a critical value for $x_{tot}^{2}$ cannot be predicted by Eq (13), which is continuous, hence the underestimation.

**Fig 5:**
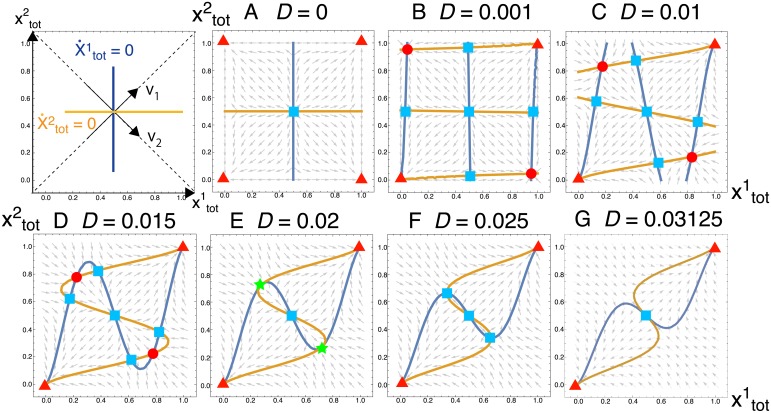
Flow diagram of Eq (10). As schematically indicated in the left-top panel, the nullclines are shown for ${\dot{x}}_{tot}^{1}=0$ and ${\dot{x}}_{tot}^{2}=0$ in blue and orange, respectively, and the crossing points of them are solutions. The directions of *v*_1_ = (1, 1) and *v*_2_ = (1, −1) are also indicated. For the solutions, stable fixed points are shown in red: those with stable growth [i.e., both fragments are in each subsystem] are in red circles, and those without growth [either of fragments is lost from subsystems or whole systems] are in red triangles. Unstable solutions are in light-blue squares, and neutral solutions in the *v*_1_-direction are in green stars at *D* = 0.02 (E). For *D* = 0 (A), the solution exists at $\left(x_{tot}^{1},x_{tot}^{2}\right)=\left(1/2,1/2\right)$ but it is unstable. For small values of *D* (B to D), the stable fixed points with growth [red circles] appear in addition to fixed points without growth. At *D* = 0.02 (E), the fixed points with growth get unstable [shown in green stars] in *v*_1_-directions. As *D* increases further (F), the two fixed points are still stable in *v*_2_-directions, while the solution at $\left(x_{tot}^{1},x_{tot}^{2}\right)=\left(1/2,1/2\right)$ is unstable in the direction. At *D* = 0.03125 (G), the system transits from the three fixed points to one fixed point.

To study further the stability of the solution, we plot the flow [a direction of the vector (${\dot{x}}_{tot}^{1},{\dot{x}}_{tot}^{2}$)] of Eq (10) in Fig 5. The steady-state solutions satisfy both ${\dot{x}}_{tot}^{1}=0$ and ${\dot{x}}_{tot}^{2}=0$, therefore, they are represented as the crossing points of two nullclines [set of $\left(x_{tot}^{1},x_{tot}^{2}\right)$ satisfying ${\dot{x}}_{tot}^{1}=0$ or ${\dot{x}}_{tot}^{2}=0$, indicated by blue and orange curves (see left-top panel)]. For *D* = 0 (Fig 5A), a solution exists at $\left(x_{tot}^{1},x_{tot}^{2}\right)=\left(1/2,1/2\right)$ (indicated by the light-blue square). However, it is unstable because the flows (arrows) point outward from the solution. Then, the system moves away from the solution by any tiny perturbation. The flows point toward each corner of the plane (indicated by the red triangles), where either of *X* or *Y* is lost and cells cannot grow. For small positive values of *D* (Fig 5B to 5D), stable fixed points (shown in red circles) appear to which the flows are directed from all directions, in addition to unstable fixed points (shown in blue squares) and the trivial solutions $\left(x_{tot}^{1},x_{tot}^{2}\right)=\left(0,0\right),\left(1,1\right)$ (shown in red triangles). Note that there exist two stable fixed points (red circles) for each *D* (Fig 5B to 5D), and the solution in Eq (12) corresponds to the right-bottom one.

As *D* increases, a bifurcation occurs at *D* = 0.02 (Fig 5E) so that the stable fixed points for *D* ≤ 0.02 turn to be unstable (green stars). To understand this bifurcation, we consider eigenvectors *v*_1_, *v*_2_ of Jacobian matrix of Eq (10) for the eigenvalues λ_1_ and λ_2_. At the stable fixed points, they are obtained as *v*_1_ = (1, 1) and *v*_2_ = (1, −1) (see left-top panel in Fig 5). The direction of *v*_1_ determines the asymmetry between *X* and *Y* in both subsystems. By moving along the *v*_1_-direction of the plane, the amount of $x_{tot}^{1}+x_{tot}^{2}$ either increases or decreases while $y_{tot}^{1}+y_{tot}^{2}=2-\left(x_{tot}^{1}+x_{tot}^{2}\right)$ decreases or increases, respectively. On the other hand, the direction of *v*_2_ corresponds to the asymmetry between subsystems 1 and 2 for the fragments of *X*. By moving along the *v*_2_-direction of the plane, the amount of $x_{tot}^{1}$ increases or decreases while $x_{tot}^{2}$ decreases or increases, respectively. The corresponding eigenvalues for *v*_1_ and *v*_2_ are calculated as $\lambda_{1}=5D-\frac{\sqrt{2D}}{2}$ and $\lambda_{2}=4D-\frac{\sqrt{2D}}{2}$, respectively. As *D* increases, a bifurcation occurs first in *v*_1_-direction at D* = 0.02 which is obtained from $5D^{*}-\frac{\sqrt{2D^{*}}}{2}=0$. In fact, the flows (arrows) at the fixed point (green stars) are in the parallel direction of *v*_2_, and point outward in the *v*_1_-directions as *D* is increased further. This corresponds to the case in which the symmetry between *X* and *Y* breaks and only either of *X* and *Y* remains in both systems. The estimated value of D* agrees with the results of our simulation (Fig 3). In the two subsystems, the bifurcation also occurs in *v*_2_-direction at *D*^+^ = 0.03125, as obtained from $4D^{+}-\frac{\sqrt{2D^{+}}}{2}=0$, corresponding to the symmetry between subsystems 1 and 2. At the bifurcation point, the three fixed points (one unstable and two stable points in *v*_2_-directions; shown all in light-blue squares) merge to one fixed point (Fig 5G).

The behavior of the bifurcations can be understood as follows. The system has two kinds of symmetry, one between fragments *X* and *Y*, and one between subsystems 1 and 2. For the stable replication, the symmetry between *X* and *Y* should be maintained because both fragments are essential. On the other hand, the symmetry between subsystems 1 and 2 should be broken because each fragment should be in excess for one subsystem, but lacking for the other subsystem. The two subsystems ‘help’ each other by the transfer of molecules. The former symmetry is maintained for 0 ≤ *D* < D* and breaks for *D* > D*. On the other hand, the latter symmetry is broken in the range 0 ≤ *D* < *D*^+^. To meet the two conditions for the stable replication, the values of *D* are restricted as 0 < *D* < D* = 0.02 because D* < *D*^+^ (*D* = 0 is eliminated by the condition each subsystem should contain both fragments).

### Frequency-dependent selection: Why the balance of fragments is achieved at the cell population

In the previous section, we confirmed the stable replication by small rates of horizontal transfer, by assuming that the populations of two cell types are equal. Here, we show that the state of an equal volume, i.e. an equal population of *X*− and *Y*−dominant cells, is stable and selected as a result of a frequency-dependent selection. To analytically investigate the stability of the state, we consider the volume fractions of the subsystems 1 and 2 are slightly different from 1/2, to be replaced by 1/2 + *ϵ* and 1/2 − *ϵ*, respectively, with *ϵ* as a small number. Then the dynamics Eqs (10) and (11) are

$$\begin{array}{c} \frac{dx_{tot}^{1}}{dt}=F^{1}-D(\frac{1}{2}+\epsilon )x_{tot}^{1}+D(\frac{1}{2}-\epsilon )x_{tot}^{2}, \end{array}$$ (14)

$$\begin{array}{c} \frac{dx_{tot}^{2}}{dt}=F^{2}-D(\frac{1}{2}-\epsilon )x_{tot}^{2}+D(\frac{1}{2}+\epsilon )x_{tot}^{1}, \end{array}$$ (15)

where the replication and the dilution terms due to the volume growth are written as $F^{i}=-x_{tot}^{i2}{\left(1-x_{tot}^{i}\right)}^{2}\left(1-2x_{tot}^{i}\right)/4$ by substituting the approximation $c^{i}=x_{tot}^{i}y_{tot}^{i}/2$.

Below, we show that the growth rate of the minor subsystem 2 with the fraction 1/2 − *ϵ* (for *ϵ* > 0) increases and the major subsystem 1 decreases, causing the fraction of the two subsystems to go back to equal. First, we write the concentrations of *X* at the steady state as $x_{tot}^{1}=x^{*}+\delta_{1}$ and $x_{tot}^{2}=1-x^{*}+\delta_{2}$ where $x^{*}=\frac{1}{2}(1+\sqrt{1-4\sqrt{2D}})$ is the solution for *ϵ* = 0 (Eq (12)), and *δ*_1_ and *δ*_2_ are deviations caused by the introduction of *ϵ*, respectively, for $x_{tot}^{1}$ and $x_{tot}^{2}$. Then, from the steady state condition of Eqs (14) and (15), one gets $\delta_{1}=\delta_{2}=\frac{\sqrt{D}\epsilon }{\sqrt{2}/2-4\sqrt{D}}$. The growth rates *μ*_*i*_ (*i* = 1, 2) are given by $\left(x_{tot}^{i}-c^{i}\right)c^{i}+\left(y_{tot}^{i}-c^{i}\right)c^{i}$ so that

$$\begin{array}{c} \mu_{1}=\mu^{*}-\gamma \left(D\right)\epsilon , \end{array}$$ (16)

$$\begin{array}{c} \mu_{2}=\mu^{*}+\gamma \left(D\right)\epsilon , \end{array}$$ (17)

where $\mu^{*}=\frac{\{1-x^{*}\left(1-x^{*}\right)\}x^{*}\left(1-x^{*}\right)}{2}$ is the growth rate at *ϵ* = 0, and $\gamma \left(D\right)=\frac{1-2\sqrt{2}D}{\sqrt{2}\sqrt{1-4\sqrt{2D}}}\sqrt{D}>0$. Eqs (16) and (17) show that the growth rate of subsystem 1 decreases with *ϵ*, whereas that of subsystem 2 increases with *ϵ*.

When *ϵ* > 0, i.e., the volume of subsystem 1 exceeds that of 2, the concentrations of *X* in both subsystems 1 and 2 increase (*δ*_1_ = *δ*_2_ > 0). For the subsystem 1, the fragment *X* is majority ($x_{tot}^{1}=x^{*}>1/2$), therefore, the asymmetry between *X* and *Y* is enhanced by the increase of *X*. On the other hand, the fragment *X* is the minority in subsystem 2, and the composition of *X* and *Y* gets close to be symmetric by *δ*_2_. Because the growth rate is maximized when the concentrations of *X* and *Y* are equal, the growth rate of subsystem 1 decreases, while that of subsystem 2 increases by the factor *γ*(*D*) > 0 (see Eqs (16) and (17)). Consequently, the volume ratio of the two subsystems eventually goes back to equal.

The above analysis indicates that the frequency-dependent selection operates if *D* > 0. However, in our simulations, the replication is unstable for small values of *D*. This discrepancy is due to the fact that discreteness in the number of molecules is taken into account in the simulations (S4 Text).

## Discussions

In summary, we have shown that the self-replication of fragmented replicases is unstable under a simple batch condition. Replication is biased towards a subset of the fragments and eventually stops due to the lack of an essential fragment. Although the stochastic correction mechanism induced by compartmentalization helps, substantial variations in the cell population are required to overcome the biased replication. This sufficient degree of variations postulates that the number of molecules *V*_*Div*_ in a cell should not be large, whereas a sufficient number of cells *N*_*cell*_ is needed. Hence, the stochastic correction mechanism imposes severe restrictions on the number of molecules per cell and the population size of cells. Then, we have shown that the horizontal transfer of intermediate frequencies provides a remedy: it gives an effective and favorable solution to the instability of the fragmented replicases. The horizontal transfer allows for the exchange of molecules between the two types of biased (*X*-dominant and *Y*-dominant) cells. Hence, this horizontal transfer relaxes the instability. Without resorting to stochastic variations, the two types of cells coexist by frequency-dependent selection. Hence, the restriction to *V*_*Div*_ and to *N*_*cell*_ is drastically reduced. The mechanism of the horizontal transfer is explained by bifurcation and frequency-dependent selection of the two deterministic subsystems. The advantage of horizontal transfer is thus demonstrated.

The relevance of horizontal transfer to a rapid spread of beneficial molecules has been discussed [18]. Here, it is importance that the horizontal transfer leads to stabilization of the replication system by sustaining each fragment, and frequency-dependent selection of two types of the cells.

Recent experimental studies have been challenged to use self-assembling fragmented ribozymes to synthesize each of the component fragments to achieve the RNA-catalyzed exponential amplification of the ribozyme itself [9]. The self-assembly of functional RNA polymerase ribozymes from short RNA oligomers has been demonstrated by driving entropically disfavored reactions under iterated freeze-thaw cycles [7]. Our theoretical results predict that these approaches for (re-)constructing RNA-based evolving systems have the serious issue: the replication of fragments is inevitably biased, so that it eventually fails to produce the copies of the ribozymes. Simultaneously, our study proposes a solution for this issue: the random exchange of fragments between loose compartments at intermediate frequencies.

Recent experiments also suggest that the random exchange of contents between compartments is plausible. The freeze-thaw cycles, which enhance the assembly of fragments [7], induce content exchange between giant unilamellar vesicles through diffusion [19]. Also, transient compartmentalization, which involves the occasional complete mixing of contents between compartments, is considered to be relevant to maintain functional replicators [20–24]. Taken together, it therefore seems natural to assume that compartmentalization is imperfect enough to allow the random exchange of fragments between compartments at the primitive stages of life.

Instead of diffusion, the mechanism of fusion and fission of vesicles is also pointed out to exchange the contents [25]. Under an appropriate rate of fusion and fission, the direct exchange of contents may avoid the system to quickly enter the regime of a well-mixed state of the whole cell population. In this case, however, there would not be a clear distinction between X-dominant and Y-dominant cells as in the present study, because the contents of the two cells are mixed completely by each event of the fusion and fission. Then, it is not obvious if the frequency-dependent selection between X-dominant and Y-dominant cells can work. Detailed investigations are needed in the future to answer whether the stabilization by the fusion and fission mechanism is as robust as that of the present horizontal transfer by diffusion.

The model of fragmented replicases investigated above can be conceptually compared to the hypercycle [26], a model proposed to solve error catastrophes: Both models posit that multiple distinct sequences are integrated into an auto-catalytic system, which as a whole maintains a greater amount of information than possible by a single sequence. However, the two models sharply differ in dynamical aspects. In the fragmented replicases, the dynamics involves the positive feedback, which biases replication toward a subset of the fragments. In the hypercycle, the dynamics involves negative feedback, which balances the replication of distinct sequences on a long timescale, but also causes oscillatory instability on a short timescale. Given these comparisons, horizontal transfer as studied here will be also relevant to hypercycles. In addition, hypercycles entail evolutionary instability due to parasites [27]. It would be interesting to study the effect of parasites on the fragmented ribozymes in the future.

While our study agrees with previous studies on the importance of compartmentalization for the maintenance of replicator diversity [14, 16, 28–31], it demonstrates a novel possibility that horizontal transfer induces negative-frequency dependent selection among replicators, a mode of selection that is known to maintain diversity in various biological systems [32]. The mechanism by which negative-frequency dependent selection arises in our model requires three elements: horizontal transfer of replicators; selection for greater replicator diversity among compartments; and positive feedback (i.e., positive-frequency dependent selection) within compartments. It remains to be investigated how generalizable the mechanism investigated here is for the maintenance of replicator diversity in prebiotic systems.

## Supporting information

S1 Text General extension of the replication to *N*-fragments ribozymes. (PDF)

S2 Text Loss of the fragments. (PDF)

S3 Text Different replication rate of the fragments *X* and *Y*. (PDF)

S4 Text Unstable growth for small transfer rate in our simulation of compartments is due to discreteness of molecules in cells. (PDF)
