# Supplementary material for: Horizontal transfer between loose compartments stabilizes replication of fragmented ribozymes
Source: PLoS Comput Biol. 2019 Jun 6;15(6):e1007094. doi: 10.1371/journal.pcbi.1007094 (PMC6581272; doi:10.1371/journal.pcbi.1007094)
Supplement: S1 Text — (PDF) [file pcbi.1007094.s001.pdf]

S1 General extension of the replication to N-fragments ribozymes

One could straightforwardly extend the two-fragments to general N-fragments system, in which N fragments of X^i $(i = 1, ..., N)$ assemble to form the catalyst. The assembly of the catalyst from the N-fragments is written as

$$X_1 + X_2 + ... + X_N \to C, \tag{1}$$

and the catalyst disassemble into the N-fragments as

$$C \to X_1 + X_2 + ... + X_N. \tag{2}$$

Each of the fragments replicates with the aid of the catalyst C as

$$X_i + C \to 2X_i + C, \tag{3}$$

for $i = 1, ..., N$.

The dynamics of the fragments X_i and the catalyst C are written as

$$\frac{dx^i}{dt} = \left(-x^1 x^2 \cdots x^N + c\right) + x^i c - x^i \mu, \tag{4}$$

and

$$\frac{dc}{dt} = \left(x^1 x^2 \cdots x^N - c\right) - c\mu, \tag{5}$$

where x^i and c denote the concentrations of X_i and C, respectively. Here, all the rate constants are fixed to one. In the right-hand-side of the equations, the terms in the brackets denote the assembly (1) and the disassembly (2) of the catalyst, and the second term in Equation (4) denotes the replication of the fragment X^i (3). Each of the last terms with μ represents dilution. The dilution terms with $\mu = (1 - Nc)c$ are introduced to fix total number of fragments as $\sum_i (x^i + c) = \sum_i x^i + Nc = 1$.

By adding both sides of Equations (4) and (5), one can write the dynamics of total concentration of X^i (total of free X^i and that in the catalyst), x^i_{tot} $(i = 1, ..., N)$ as

$$\frac{dx^i_{tot}}{dt} = (x^i_{tot} - c)c - x^i_{tot}\mu,$$

where the concentration of the free X_i, x^i, is written as $x^i_{tot} - c$. Then, for arbitrary pair of i and j $(i, j = 1, ..., N)$, one obtains

$$\frac{d}{dt}\left(\frac{x^i_{tot}}{x^j_{tot}}\right) = \frac{c^2}{x^{j2}_{tot}}\left(x^i_{tot} - x^j_{tot}\right) \tag{6}$$

Given that $(c/x^j_{tot})^2$ is positive, this indicates that a small increase of x^i_{tot} over x^j_{tot} grows so that the solution is unstable of balanced replication $x^i_{tot} = x^j_{tot}$ for every pair of i and j.
