# Supplementary material for: Horizontal transfer between loose compartments stabilizes replication of fragmented ribozymes
Source: PLoS Comput Biol. 2019 Jun 6;15(6):e1007094. doi: 10.1371/journal.pcbi.1007094 (PMC6581272; doi:10.1371/journal.pcbi.1007094)
Supplement: S2 Text — (PDF) [file pcbi.1007094.s002.pdf]

S2 Loss of the fragments

All the fragments that diffuse out of a cell are added to one of the other cells. Thus, the fragments are not lost in our simulation presented in the main text. The fragments that diffuse out of a cell, however, can be actually lost from the system.

We show that our results are not sensitive to a partial loss of the fragments. To demonstrate this, we extend the simulation to implement the loss of the fragments. We assume that the fragments that diffuse out of a cell are lost with a probability p, thus, they are not added into other cells. The stabilization by the horizontal transfer is largely valid in this case and different cells can compensate for one another's imbalances [Figure S1]. With increasing p, however, the asymmetry between the major and minor fragments gets larger because the rate of receiving minor fragments decreases by the loss.

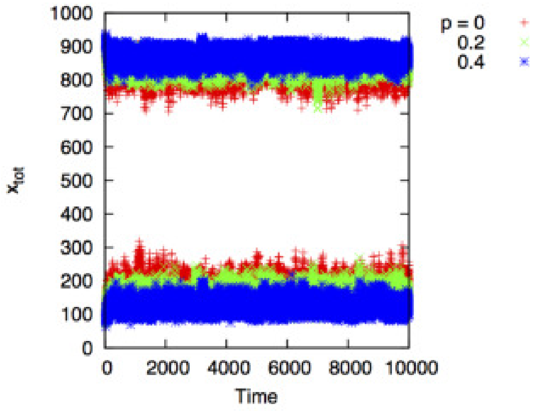

Figure S1: The number of fragments X_{tot} of dividing cells. With a probability p, the fragments that diffuse out of a cell are lost and they are not added into other cells. The parameters are $D = 0.01$, $V_{Div} = 1000$, $N_{cell} = 100$, $k^f = k^b = 1$, $k_x = k_y = 1$.
