# Supplementary material for: Horizontal transfer between loose compartments stabilizes replication of fragmented ribozymes
Source: PLoS Comput Biol. 2019 Jun 6;15(6):e1007094. doi: 10.1371/journal.pcbi.1007094 (PMC6581272; doi:10.1371/journal.pcbi.1007094)
Supplement: S3 Text — (PDF) [file pcbi.1007094.s003.pdf]

S3 Different replication rate of the fragments X and Y

In the main text, we assume that the replication rates k_x and k_y are the same in the reactions (2) and (3). Here, we show that the mechanism of the horizontal transfer apply even when the k_x and k_y are different, provided that there is not a large difference. In this case, the ratio of the number of X-dominant and Y-dominant cells change to compensate for the difference of k_x and k_y [Figure S2]. When k_x is greater than k_y [Figure S2A], the number of X-dominant cells decreases and that of Y-dominant cells increases. On the other hand, when k_x is smaller than k_y [Figure S2B], the number of X-dominant cells increases. When the difference increases $\approx 20\%$ or larger, a faster-replicating fragment dominates the system and the system cannot continue growth [Figure S2C].

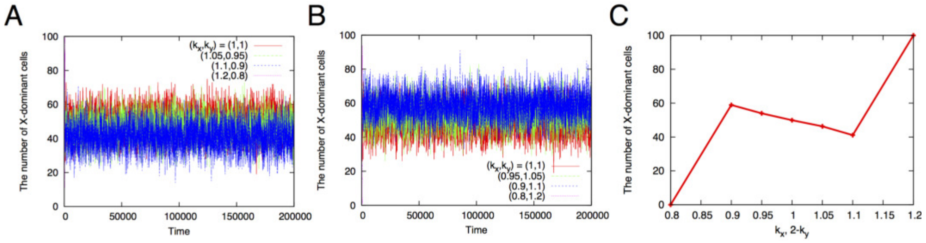

Figure S2: The number of X-dominant cells for (A) $(k_x, k_y) = (1,1), (1.05, 0.95), (1.1, 0.9), (1.2, 0.8)$ and (B) $(k_x, k_y) = (1,1), (0.95, 1.05), (0.9, 1.1), (0.8, 1.2)$. (C) The average of the number of X-dominant cells is shown as a function of k_x. The parameters are $D = 0.001$, $V_{Div} = 1000$, $N_{cell} = 100$, and $k^f = k^b = 1$.
