# Supplementary material for: Horizontal transfer between loose compartments stabilizes replication of fragmented ribozymes
Source: PLoS Comput Biol. 2019 Jun 6;15(6):e1007094. doi: 10.1371/journal.pcbi.1007094 (PMC6581272; doi:10.1371/journal.pcbi.1007094)
Supplement: S4 Text — (PDF) [file pcbi.1007094.s004.pdf]

S4 Unstable growth for small transfer rate in our simulation of compartments is due to discreteness of molecules in cells

The bifurcation analysis presented in the main text explains that the system is stable for infinitely small but non-zero values of D for the two subsystems. On the other hand, the system gets unstable in our simulation of compartments for small non-zero values of D, because the system is dominated by either of the X- or Y-dominant cells and the symmetry between X and Y is broken.

As D decreases, the system is dominated by either of the cells because fluctuations increase between the number of X- and Y-dominant cells and, once the number of either type of cells reaches the population size N_{cell}, it irreversibly breaks the symmetry between X and Y. Actually, the variances σ^2 of the number of X-dominant cells increases as D decreases [Figure S3A].

The increase of the variances suggests that pressure of the frequency-dependent selection to maintain the symmetry is getting weak as D decreases. However, the analysis up to the first order of ϵ presented in the main text does not explain such dependence on D for the two subsystems. The pressure to maintain the symmetry is represented by relative values of the factor $\gamma(D)$ to the average growth rate μ^* in Equations 16 and 17 of the main text. If the relative values decrease with decreasing D, the pressure would be weakened because the relative increase or decrease of the growth rates, respectively, between subsystems of smaller or larger volumes gets smaller. Actually the factor $\gamma(D)$ depends on D as $\sqrt{D}$ for small D [see Figure S4], but the average growth rate μ^* also scales as $\sqrt{D}$. Therefore, the relative pressure of the frequency-dependent selection does not change with decreasing D.

This suggests that the unstable growth in our simulation of compartments is due to the discrete nature of molecules in cells because the effect is neglected in the two subsystems. As D decreases, the number of minor fragments decreases [Figure 4 of the main text], therefore, the cells gradually contain only a few or no catalyst. In fact, the number of cells with the catalysts C gradually decreases with decreasing D [Figure S3B: the case for both X/Y-fragments and catalysts also shows similar curves] in accordance with the increase of the variances σ^2 of the number of X-dominant cells [Figure S3A]. Therefore, the discrete nature of molecules in cells contributes to the enhancement of variations between cells and consequently results in the increase of the variances.

A scaling behavior between the transfer rate D and the division threshold V_{Div} is also consistent with the enhancement of the variances by the discreteness of molecules. The relevant values of the transfer rate D with which the effect of the discrete number of molecules appears scales as $1/V_{Div}^2$. For substantially small D, the concentration of minor fragments in Equation 13 of the main text can be written as $x_{tot}^1 = \sqrt{2D}$. Therefore, the values of D at which the total number of minor fragments is approximately equal to one scale as $D \approx 1/2V_{Div}^2$. In fact, the numbers of cells with X fragments and catalysts C and the variances for different V_{Div} and N_{cell} agree as one plot as a function of DV_{Div}^2 [Figure

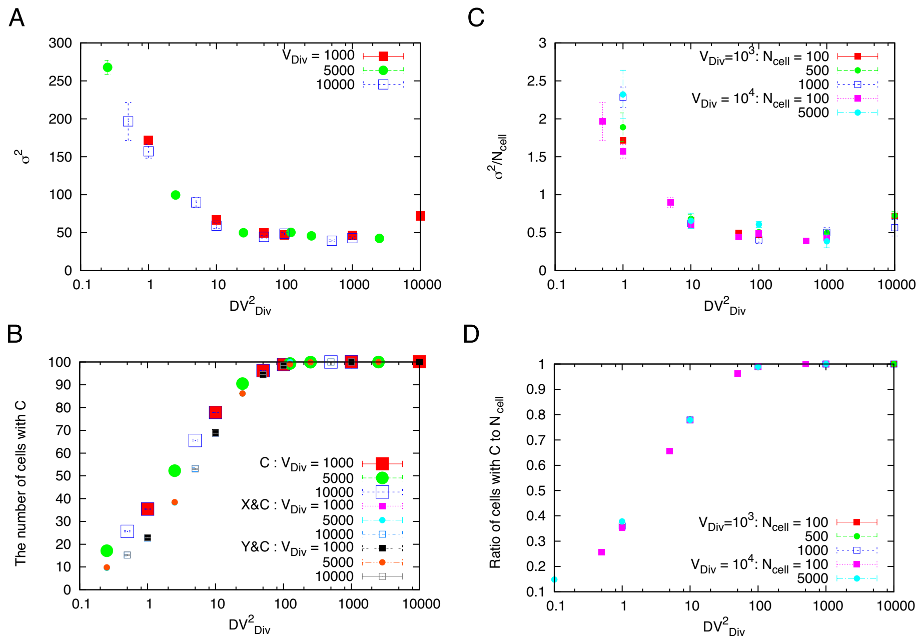

Figure S3: (A) Variances σ^2 of the number of X-dominant cells as functions of D and V_{Div}. Here, $N_{cell} = 100$. (B) The number of cells with catalysts C scales with DV_{Div}^2 for $V_{Div} = 1000, 5000, 10000$. The numbers of cells with both C and free X/Y also show a similar curve. Here, $N_{cell} = 100$. (C) Variances σ^2 of the number of X-dominant cells divided by N_{cell} and (D) Ratio of cells with the catalysts C to N_{cell} are shown for different values of N_{cell}. All the points follow the same curve under the scaling DV_{Div}^2.

S3C and D]. This suggests that, when V_{Div} is infinitely large, the variances do not increase even when D decreases to infinitely small.

Further, the system is stable if the number of cells N_{cell} is large because the variances of the ratio of X-dominant to Y-dominant cells scales as $1/\sqrt{N_{cell}}$ with N_{cell}. The variances σ^2 of the number of X-dominant cells divided by N_{cell} collapses into the same curves for different N_{cell} [Figure S3D]. This indicates that they increases with $\sqrt{N_{cell}}$, and the ratio of X-dominant cells to total population scales as $1/\sqrt{N_{cell}}$. Thus, fluctuations in the ratio of X-dominant to Y-dominant cells decreases when N_{cell} is getting large.

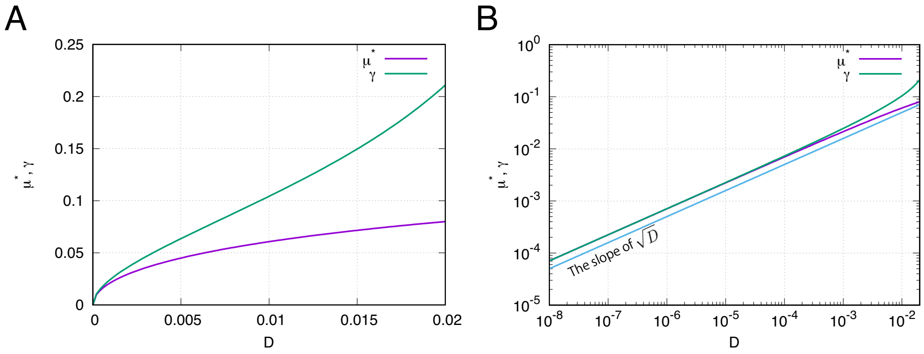

Figure S4: Growth rate μ^* and $\gamma(D)$ of Equation 16 and 17 of the main text as a function of D in (A) normal and (B) log-log scale for small D. The slope $\sqrt{D}$ is also shown in (B).
